# Detailed Characteristics of Tonsillar Tumors with Extrachromosomal or Integrated Form of Human Papillomavirus

**DOI:** 10.3390/v12010042

**Published:** 2019-12-30

**Authors:** Barbora Pokrývková, Martina Saláková, Jana Šmahelová, Zuzana Vojtěchová, Vendula Novosadová, Ruth Tachezy

**Affiliations:** 1 Department of Genetics and Microbiology, Faculty of Science, Charles University, BIOCEV, Průmyslová 595, 25250 Vestec, Czech Republic; barbora.pokryvkova@natur.cuni.cz (B.P.); jana.smahelova@natur.cuni.cz (J.Š.); zuzana.vojtechova@natur.cuni.cz (Z.V.); tachezr@natur.cuni.cz (R.T.); 2 Czech Centre for Phenogenomics, Institute of Molecular Genetics of the Czech Academy of Sciences, BIOCEV, Průmyslová 595, 25250 Vestec, Czech Republic; vendula.novosadova@img.cas.cz

**Keywords:** human papillomavirus, E2 binding sites, methylation, genome status

## Abstract

The human papillomavirus (HPV) integration, the critical step in viral carcinogenesis, most frequently occurs in the E2 gene, which results in its inactivation and in an increase of E6/E7 transcription. However, in a substantial number of tumors, the virus is present in an extrachromosomal form. For those tumors, the transformation mechanisms are not fully elucidated. Here we evaluated the possible mechanism of inactivating the E2 without interruption of the gene, methylation or mutation of the E2 binding sites (E2BSs) in HPV16-positive tonsillar tumors by next-generation and Sanger sequencing. Viral genome status was analyzed by the amplification of papillomavirus oncogene transcripts assay (APOT) and mRNA mapping, and expression of viral oncogenes was performed by qPCR. The methylation of E2BSs was significantly higher in tumors with an integrated, in comparison to extrachromosomal, form of the viral genome. No mutations were detected in the E2BSs. The viral oncogenes were equally expressed in samples with an integrated and extrachromosomal form of the virus. Only the nucleotide variants were identified in the E2 gene. No proposed mechanism of E2 inactivation was confirmed in tonsillar tumors with an extrachromosomal form of the HPV genome. The expression of E6/E7 genes seems to be sufficient to initiate and maintain the carcinogenic process

## 1. Introduction

Every year, more than 650,000 new cases of head and neck carcinomas (HNCs) are diagnosed worldwide [1]. Most HNCs are associated with smoking and alcohol consumption, but nearly 26% of HNCs are linked to infection by high-risk human papillomavirus (HR HPV) [2]. HPV-driven HNCs are mostly found in the oropharynx, with HR HPV prevalence varying from 7% to 88%, according to the sub-site of oropharyngeal cancer (OPC), the detection method and geographical area [3]. The incidence of OPC has been increasing markedly over the last decade [4,5], and it is widely accepted that HPV-associated HNC represents a different entity. From the clinical point of view, the most important are the improved survival and response to treatment in HPV HNC patients [6].

The essential role in the process of viral carcinogenesis is played by two viral oncoproteins, E6 and E7. These viral oncoproteins, via binding to the cellular targets p53 and pRb, cause disruption of cellular growth control pathways [7]. HPV16–E6/E7 oncogene transcription is initiated from the viral P97 promoter located in the non-coding long control region (LCR), and is fundamentally regulated by the viral E2 protein. 

The E2 protein binds to four specific binding sites (E2BS1-4) with a consensus palindromic DNA motif (ACCGN_4_CGGT). In the early stages of the viral infection, low-abundance E2 binds to its high-affinity site (E2BS1), which activates viral oncogene transcription and maintains E2 expression. Increasing E2 levels lead to its binding to the repressive low-affinity sites (E2BS3-4) to suppress viral oncogene transcription. The function of E2BS2 is still uncertain (reviewed in Doeberitz and Vinokurova, 2009, McBride and Warburton, 2017 [8,9]).

In the permissive viral life cycle, the HPV genome is maintained in an extrachromosomal form. During persistent infection, the HPV genome may integrate into the host genome at fragile and/or transcriptionally-active sites without chromosomal preference. Regarding the viral integrated genome, it is frequently, but not exclusively, interrupted in the E2 ORF, which leads to E2 inactivation. It has been reported that E2 loss results in the disruption of the negative feedback E2 on the E6/E7 oncogene transcription [10]. The Cancer Genome Atlas (TCGA) analysis of 279 HNC samples revealed also high frequencies of breakpoints in the E1 gene [11]. As documented from premalignant cervical lesions and cervical cancer (CC), the proportion of cells harboring the integrated genome increases with the disease progression [12], and patients with the integrated HPV genomes have worse survival rates in comparison to those with the extrachromosomal form of the virus [13,14,15]. In patients with OPC, the presence of an intact E2 ORF was associated with improved clinical outcome in comparison to that in patients with tumors with a disrupted E2 gene [16,17], however in larger set of patients, no statistically significant difference was found in disease-specific survival between patients with integrated vs. extrachromosomal and/or mixed forms of HPV [18]. On the other hand, up to 45% of CC and 51% of HNC tumors harbor only extrachromosomal HPV genomes [12,18,19,20], which demonstrates that integration is a frequent but not necessary step in HPV-initiated cancer progression.

Disruption of the E2 gene has been correlated with a higher expression of viral E6/E7 mRNA in the cervical cancer cell line [21,22], but the results obtained from patients’ samples of CC as well as OPC are inconsistent with in vitro observation [19,23,24]. The viral load has been shown to correlate with disease severity and E6 and E7 oncogene expression in cervical cancer [25,26]. However, in HNC and HNC-derived cell lines, no association was found between viral load, integration and viral oncogene expression [23,27].

Epigenetic modifications have been proposed as another possible mechanism for E2 inactivation [28,29,30]. CpG methylation at the repressive E2BSs may disable E2 binding and prevent its repressive function on E6/E7 oncogene transcription. The methylation levels of the E2BS1, -3 and -4 were found to be higher in CC with extrachromosomal compared to integrated HPV genomes [28]. This phenomenon has also been observed for the repressive E2BSs in HPV-driven oropharyngeal cancers [31]. Another possible mechanism of disrupting the negative feedback of E2 in E6/E7 transcription could be mutations in the repressive E2BS3 or E2BS4 that make this regulation ineffective. 

In this study, we searched for the mechanism by which HPV16 present in the tonsillar carcinoma tissues in an extrachromosomal form contributes to the malignant transformation of the cell. We compared the methylation levels, mutation frequencies in E2BSs, presence of nucleotide variants in LCR and the complete E2 gene, viral load and expression levels of viral oncogenes E6 and E7 in HPV-driven tonsillar carcinomas with the extrachromosomal or integrated virus genomes.

## 2. Materials and Methods 

### 2.1. Clinical Samples

Samples of primary squamous cell carcinomas of the oropharynx (ICD-10:C09, C099, and C091) were obtained from the Department of Otorhinolaryngology and Head and Neck Surgery, 1st Medical Faculty, Charles University and Motol University Hospital, Prague. They were collected in 2001–2007 within our previous studies. Detailed characteristics of patients and samples have been described previously [18,32]. Sections of the primary tumor were fixed by a pathologist with tissue freezing medium, frozen in liquid nitrogen, and stored at −80 °C. The other sections of each tumor were fixed in 10% neutral formalin and paraffin embedded (FFPE). 

The first and the last sections were histologically confirmed by the pathologist and carefully evaluated for the presence and percentage of dysplastic/tumor cells. Eighteen tonsillar carcinoma samples were selected for this study, including HPV16-positive samples with active viral infection from the study of Rotnáglová et al. (2011) [32]. 

### 2.2. Processing of Samples and Detection of an Active Infection

DNA and total RNA from samples was isolated using the QIAamp DNA Mini Kit (Qiagen, Hilden, Germany) and miRVana Kit (Life Technologies, Carlsbad, CA, USA), respectively. The concentration was measured by a NanoDrop 2000 spectrophotometer (ThermoFisher Scientific, Waltham, MA, USA). The presence of HPV16 infection was confirmed by the broad spectrum GP5+/GP6+-5’bio PCR assay and reverse line blot analysis, as described previously [33].

A subset of 12 fresh frozen tissue samples were analyzed in our previous study for the presence of active HPV infection by the detection of HPV16–E6 mRNA [18]. Additional six samples were newly isolated and characterized within this study. 

### 2.3. HPV Genome Status Determination

The HPV genome status was evaluated by a modified amplification of papillomavirus oncogene transcripts (APOT) assay and by mapping of HPV E2 mRNA breakpoints as described by Vojtechova et al. (2016) [18] (Supporting Information Table S1). Shortly, in the APOT assay, RNA was reverse transcribed using adaptor-linked oligo (dT)_17_ primer, the cDNA was then amplified by nested polymerase chain reaction (PCR) using an HPV16–E7-specific primer and linker for the first run and second E7-specific primer and adaptor-linked oligo (dT)_17_ primer for the second run of the PCR. The amplicons were separated on 2% agarose gel and blotted on a nylon membrane (PerkinElmer, Waltham, MA, USA) using alkaline transfer and then hybridized with HPV16–E7- and E4-specific probes labelled with biotin. The hybridized probes were visualized by streptavidin-horseradish peroxidase (HRP) followed by chemiluminescent detection by the electrochemiluminescence (ECL) kit (GE Healthcare, Chicago, IL, USA). All amplicons longer than 300 bp were excised from the gel and sequenced using the BigDye Terminator Sequencing Kit v 3.1. (Applied Biosystems, Foster City, CA, USA). In addition to the APOT assay, E2 integration breakpoint mapping was done on cDNA using the set of primers amplifying E7, E2, E4 and E5 mRNA [18]. The products of amplification were visualized on 3% agarose gel. 

### 2.4. Methylation Analysis

Three hundred nanograms of DNA of 18 samples underwent bisulfide treatment using the EZ DNA Methylation™ Kit (ZYMO Research, Irvine, CA, USA) according to the manufacturer’s instructions. The treated DNA was subjected to the two different nested PCR assays using primer pairs covering the E2BS1 and E2BS2-4 of the HPV16 LCR. In the first round of PCR, the specific set of primers designed for this study with a key adaptor sequence (Forward: CACGACGTTGTAAAACGAC-, Reverse: CAGGAAACAGCTATGACC-) for a unique barcode sequence (MID) were used to amplify the 163 bp region of the distal E2BS1 with two CpGs (Forward: adaptor-TTGTTTTAATATTTATTAATTGTGTTGTG; Reverse: adaptor-AAACTATTTAAAAAAACACATTTTATACC). For E2BS2-4, a set of primers which covered the 179 bp region with the three E2 binding sites designed by Chaiwongkot et al. (2013) was used with the adaptor sequences extended [28]. These two reactions consisted of 1× ZymoTaq™ Reaction Buffer with MgCl_2_, 0.25 mM of each dNTP, 2 U of Hot Start ZymoTaq™ DNA polymerase (ZYMO Research, Irvine, CA, USA), 0.12 µM of each primer (adaptor-E2BS1), or 0.4 µM of each primer (adaptor-E2BS2-4), respectively, in a total volume of 25 µL. Both reactions were performed in a Peltier Thermal Cycler 200 (MJ-Research, Waltham, MA, USA). Conditions were as follows: 95 °C for 10 min, followed by 50 cycles of 95 °C for 30 s, 48 °C for 40 s and 72 °C for 40 s, with a final extension of 72 °C for 7 min. One microliter of amplicons was transferred to the second round of PCRs with primers specific to the key adaptor sequence and tailed with MID. 

The reactions contained 1× ZymoTaq™ Reaction Buffer with MgCl_2_, 0.5 mM of each dNTP, 2 U of Hot Start ZymoTaq™ DNA polymerase (ZYMO Research, Irvine, CA, USA), and 0.2 µM sample-specific MID-tailed primers. The amplification reaction was performed in a Peltier Thermal Cycler 200 (MJ-Research, Waltham, MA, USA) under the following conditions: 95 °C for 10 min, followed by 35 cycles of 95 °C for 30 s, 60 °C for 40 s and 72 °C for 1 min, with a final extension of 72 °C for 10 min. The amplified PCR products were purified using Agencourt AMPure XP magnetic beads (Beckman Coulter, Brea, CA, USA), quantified using Qubit 2.0 (Thermo Fisher Scientific, Waltham, MA, USA), and equimolar mixed. One hundred thousand molecules of this mixture were used as a template for the emulsion PCR performed using the GS Junior Titanum emPCR Lib-A Kit (Roche, Indianopolis, IN, USA). Pyrosequencing was done by the GS Junior Titanium Sequencing Kit on a GS Junior apparatus (Roche, Indianopolis, IN, USA) according to the manufacturer’s instructions. For each amplicon, at least 200 reads were analyzed for the methylation status (methylated/unmethylated) using the GS Amplicon Variant Analyzer (Roche, Indianopolis, IN, USA) and QUMA online software (http://quma.cdb.riken.jp).

### 2.5. HPV16 DNA Load

The viral load of HPV16 was determined in triplicates by qPCR with HPV16–E6-specific primers and probes [34] using the RotorGene 6000 (Qiagen, Hilden, Germany). The qPCR assay for the single copy human RNaseP gene was used to normalize HPV16 viral copy numbers to cell numbers, as previously described [35]. The calculation of initial copy numbers in samples was performed by the RotorGene 6000 software (Qiagen, Hilden, Germany), using a standard curve generated for the quantification of ten-fold dilution series standards, plasmid with full length HPV16, and pCR-BluntII-TOPO vector (Invitrogen, Carlesbad, CA, USA) with cloned RNaseP insert. Final viral loads of all samples were normalized using normalization factor (NF)*dysplastic cells fraction (DCF) as follows: viral load of samples with a <10%, 25%, 50%, 75% and >90% DCF were multiplied by NFs of 10, 4, 2, 1.5, and 1, respectively [24].

### 2.6. HPV16 Oncogene E6/E7 mRNAs Quantification

We quantified the expression levels of two E6/E7 transcripts by the RT-qPCR method using SYBR Green chemistry, as described before [24]. Both products are initiated from the P97 promotor; the spliced E6*I transcript corresponds to the E7 protein (E6*I) and the second product is the unspliced, full length E6/E7 transcript (E6 FL). The detection of the reference HPRT gene [23] was used for the normalization of the mRNA level as well as the formula regarding DCF as described above. First, 2 µg of DNase-treated total RNA were reverse transcribed using M-MLV Reverse Transcriptase (Promega, Madison, WI, USA) according to the manufacturer’s protocol. qPCR experiments were carried out on the RotorGene 6000 system (Qiagen, Hilden, Germany) in triplicates. The 15 µL reaction contained 1× AmpliTaq Gold PCR buffer II, 4 mM MgCl_2_ (both Applied Biosystems, Foster City, CA, USA), 0.2 mM dNTPs (Promega, Madison, WI, USA), 0.5 µM of each primer, 25,000× SYBR^®^Green I dye (Molecular Probes, USA), 1 U of AmpliTaq Gold DNA polymerase (Applied Biosystems, Foster City, CA, USA) and 1 µL of 5× diluted cDNA. The reaction conditions for all genes were as follows: 50 °C for 2 min, 95 °C for 10 min, followed by 40 cycles of 95 °C for 15 s, 60 °C for 30 s, 72 °C for 30 s, and the melting curve. The amplification efficiency of all assays was evaluated using the standard curve from the ten-fold serial dilutions of the pCR-BluntII-TOPO plasmid with cloned target sequences (10^7^–10^1^ copies/µL). The data processing and quantification of mRNA expression were performed with the RotorGene 6000 software, v. 1.7 (Qiagen, Hilden, Germany).

### 2.7. LCR and E2 Variant Analysis

To detect the variants in E2BSs, viral LCR was amplified with V16C/V16D primers covering the 682 bp region, as described earlier [36,37]. For E2 variant detection, the whole E2 gene (1167 bp region) was amplified, as published before [38]. Amplicons were excised from the gel and directly sequenced using the BigDye™ Terminator Sequencing Kit v. 3.1 on the ABI 3500 instrument (both Applied Biosystems, Foster City, CA, USA) using specific outer and inner primers [37,38]. 

Sequence data were analyzed using the BLAST software (http://www.ncbi.nlm.nih.gov/BLAST) and compared both with the HPV16 reference sequence (GenBank: K02718.1) and HPV16 Asian-American variant (D3 lineage) sequence (GenBank: AF402678.1) from PAVE database (https://pave.niaid.nih.gov/) using LaserGene software (DNASTAR, Madison, WI, USA).

### 2.8. Statistical Analysis

The CpG methylation levels in the groups of samples divided according to the viral genome status were calculated from the numbers of methylated and unmethylated reads for all four E2BSs. Data were analyzed in R (package LmerTest). We tested for any difference between groups using the general mixed model with random effect ID of sample nested in Position. Data obtained from the viral load and mRNA expression analysis were first tested for the normality using the Shapiro-Wilk normality test and analyzed by the nonparametric Mann-Whitney *U*-test. Spearman’s rank correlation coefficient between the number of integrated copies and the methylation level was determined. Statistical analyses were performed by GraphPad Prism 6 (GraphPad Software, San Diego, CA, USA). 

## 3. Results

### 3.1. HPV E2 mRNA Determination

The E2 breakpoint mapping of viral mRNA covered the E7, E1, E2, E4 and E5 ORFs and revealed the intact, complete viral E2 gene in 13 samples (13/18, 72.2%). These transcripts were considered to originate from extrachromosomal HPV genomes. In the remaining five samples, no E2 complete mRNA was detected (5/18, 27.8%), which predicted the integration and disruption in E2 ORF. In these samples, only E7 mRNA was detected with the exception of one sample (ORL 125), where part of E2 mRNA was present. 

The APOT assay identified the standard extrachromosomal transcript with a length of 1050 bp (E7–E1^E4–E5) in 13 samples (13/18, 72.2%), and integrated E6–E7-cell fusion transcripts in eight samples (8/18, 44.4%). Four samples (4/18, 22.2%) contained both integrated fusion transcripts as well as the standard extrachromosomal transcript. The integrated viral transcripts were spliced from the viral splice donor (SD) 880 to the cellular splice acceptor (SA) sites (transcripts type A, [39]). For one sample (ORL 128), using the APOT assay, only short, non-spliced E6/E7 transcripts were identified, while E2 breakpoint mapping detected only the E7 mRNA and no other mRNAs, and the sample was marked as integrated. Based on the results, 50% of samples (9/18) contained the extrachromosomal form of the HPV16 genome, 22.2% (4/18) of samples a mixed (integrated fusion transcripts as well as the standard extrachromosomal transcript) form, and 27.8% (5/18) an integrated form of the HPV genome according to both methods used. The samples were grouped according to the presence and stable expression of complete E2 mRNA and viral–host fusion transcripts in two groups; group one represents samples with an expression of the E2 gene, the potential E6/E7 transcription repressor, as well as E4/E5 genes according to E2 breakpoint mapping and APOT assay, and thus samples with the extrachromosomal or mixed form of HPV, and group two with samples harboring an integrated form of HPV, which contained E6–E7-cell fusion transcripts only, and no E2 mRNA. The whole E2 gene was present in the majority of samples (16/18, 88.9%) irrespective to the detection of viral E2 mRNA and/or viral–host fusion transcripts. Three samples with integrated form with the E2 gene showed no E2 mRNA transcription. All results are shown in Table 1.

### 3.2. Methylation Analysis

**Figure 1:**
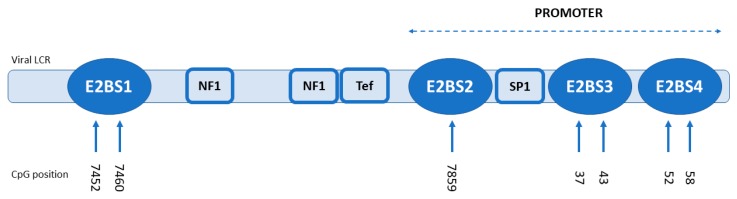
CpG nucleotide positions in the HPV16 long control region (LCR), as compared with the HPV16 reference sequence (GenBank: K02718.1).

**Figure 2:**
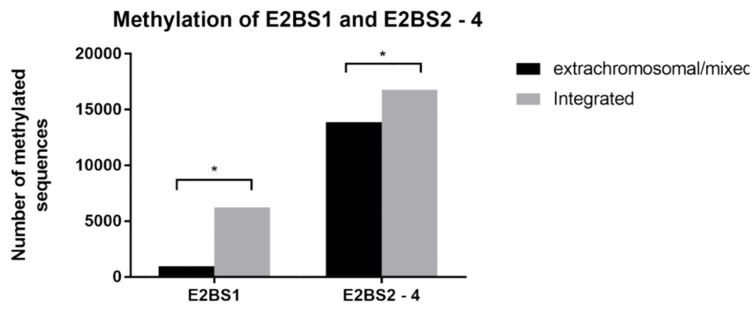
CpG methylation levels of activating E2BS1 and repressive E2BS2-4. Numbers of methylated sequences were compared between samples with extrachromosomal/mixed or integrated viral genomes both for activating E2BS1 and repressive E2BS2-4. The difference was statistically significant for both areas (*p* = 0.028 for E2BS1 and *p* = 0.034 for E2BS2-4).

To detect and quantify the CpG methylation level of four E2BSs in HPV16 LCR, targeted bisulfite pyrosequencing was performed. Detailed results and numbers of methylated or unmethylated CpGs detected in all four E2BSs are shown in Table 2. One sample (ORL104) was excluded before analysis due to presence of lineage D of HPV16 and the unsuccessful E2BS amplification. The coverage of two amplicons, the E2BS1 amplicon of the ORL 187 sample and E2BS2-4 of sample ORL 125, was lower than 200 reads; therefore, they were also excluded from the analysis. For all other amplicons, an average overall coverage of 2261 reads was obtained (the number of reads ranged from 271 to 3270 between amplicons). In total, seven CpGs were analyzed and compared with the HPV16 reference sequence (GenBank: K02718.1): two for activating the E2BS1 area (nucleotide positions 7452 and 7460), four for repressive E2BS3-4 (nucleotides position 37, 43, 52 and 58), and one for E2BS2 (nucleotide positions 7859) (Figure 1). The repressive E2BS2-4 area and activating E2BS1 area exhibited statistically significantly higher methylation levels in the samples with integrated DNA in comparison to those with the extrachromosomal form (*p* = 0.034 for E2BS2-4 area and *p* = 0.028 for E2BS1) (Figure 2). In our group of samples, we have noticed a much higher methylation level at repressive E2BS2-4 sites compared to E2BS1 regardless of the viral genome status. 

### 3.3. HPV Viral Load Analysis

**Figure 3:**
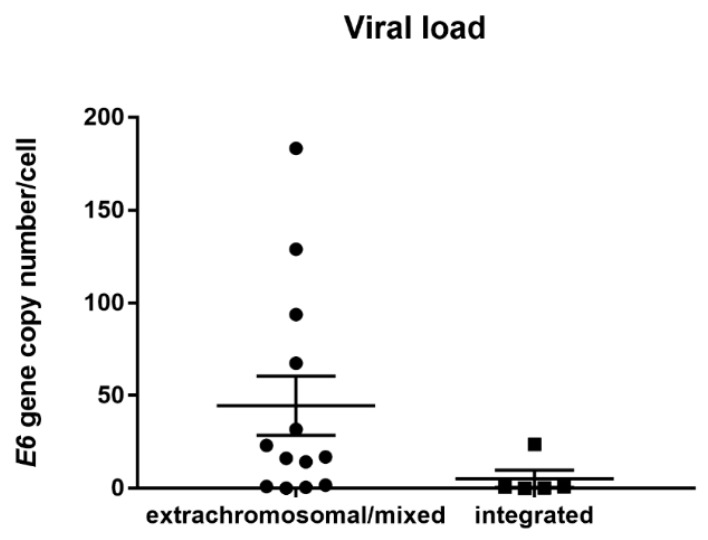
HPV16 viral load evaluation. Comparison of E6 gene copy numbers relative to RNase P copy number in tumors with extrachromosomal/mixed and integrated form of the viral genome. The difference was not statistically significant (*p* = 0.075).

To estimate the HPV16 copy number in the samples with integrated vs. extrachromosomal/mixed forms of the viral genome, qPCR was performed. HPV16 viral load ranged from <1 to 183 copies per cell, but there was no statistically significant (*p* = 0.075) difference between samples with the extrachromosomal/mixed and integrated form of the genome (range 2 × 10^−2^ to 183, average copy number 49 per cell and median 16.84; range 2 × 10^−1^ to 23, average copy number five per cell, and median 0.65, respectively) (Figure 3). Furthermore, we observed no correlation between the viral load and methylation level in the repressive E2BS2-4 region (Spearman rank correlation, *r* = 0.400, *p* = 0.750 for integrated genome samples and *r* = 0.175, *p* = 0.588 for extrachromosomal/mixed genome samples).

### 3.4. Quantification of Viral E6/E7 Oncogene Transcripts

**Figure 4:**
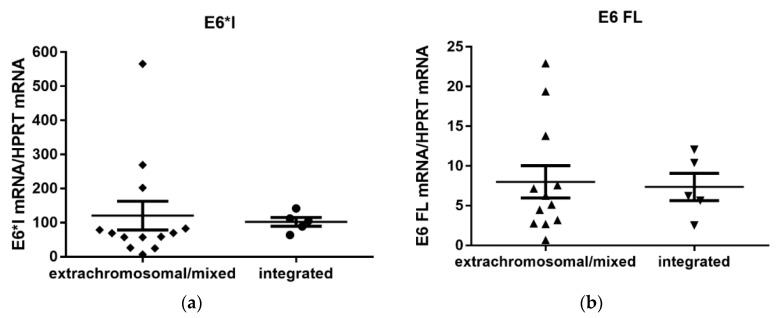
E6/7 oncogene transcription levels. Number of spliced E6 transcripts (E6*I, **a**) and full length E6 transcripts (E6 FL, **b**) in samples with extrachromosomal/mixed and integrated HPV16 genomes normalized to HPRT mRNA. The difference is not statistically significant (*p* = 0.208 for E6*I and *p* = 0.959 for E6 FL).

The more abundant of the two HPV16 transcripts was the E6*I, while the E6/7 FL transcript showed a lower expression level (Figure 4). The transcription level of the two oncogene transcripts did not differ significantly between the samples with the integrated and extrachromosomal/mixed form of the viral genome (*p* = 0.208 for E6*I mRNA, and *p* = 0.959 for E6/7 FL mRNA, respectively). We further evaluated if there was a correlation between the mRNA expression of the E6*I transcript and viral load, both in samples with the extrachromosomal/mixed and integrated viral genome. No correlation was observed for either extrachromosomal/mixed genome samples or integrated genome samples (*r* = −0.258, *p* = 0.394; *r* = 0.3, *p* = 0.683, respectively).

To verify the results of previous studies, the effect of the methylation status on viral transcription was also investigated. Again, there was no correlation between the average percentage of the methylated sequences of repressive E2BS2-4 and E6*I mRNA levels for the two groups of samples with different forms of the viral genome (Spearman rank correlation, *r* = 0.077, *p* = 0.817 for extrachromosomal/mixed genome samples and *r* = −0.6, *p* = 0.417 for integrated genome samples). 

### 3.5. LCR and E2 Variants Analysis

Viral LCR of 18 samples was sequenced to detect mutations in E2BSs. Except for sample ORL 104, which matches with the D lineage variant, all samples belonged to the most prevalent A variant lineage. In these A variant samples, 11 different sequences variations were detected, but none of them was localized at the E2BSs. The most abundant polymorphism was the G to A transition at position 7518, detected in 13/17 samples, and corresponding to a YY1 transcription factor binding site (Table 3**)**. We evaluated the E6*I mRNA level in samples with sequences variation at position 7518 (*N* = 13) and those with a wild type nucleotide (*N* = 4), and found no differences in transcription level (*p* = 0.0597). We also detected variants of the E2 gene. Altogether we detected 12 polymorphisms which resulted in 9 amino acid changes, but no variants result in protein truncation (Supporting Table S2). 

## 4. Discussion

Integration of the HPV genome into the cell genome is an important but not essential step in virus-driven carcinogenesis in cervical and head and neck carcinomas. The integration imparts a selective growth advantage through an enhanced expression and higher stability of fusion virus–host transcripts [10]. Also, it seems that particular cell clones are selected during the transformation process according to the site of integration. These clones are characterized by increased proliferation and improved survival [11]. HPV integration itself is directly associated with genetic instability and extensive host genomic amplifications and rearrangements [40]. However, in many HPV-driven tumors, the integration of viral genomes into host chromosomes were not detected, and the cancer initiation mechanism is still not fully elucidated. To clarify the mechanisms of carcinogenesis driven by the extrachromosomal form of HPV as well as by the mixed form with expression of E2, we performed complex analyses of 18 tonsillar tumor samples with HPV16 active infection. LCR and E2 ORFs single nucleotide variant analysis, determination of CpG methylation level of E2BSs, viral load evaluation and viral oncogene E6/E7 transcript quantification, were done. The E2 mRNA detection using mapping of the viral transcripts was chosen as the selective marker for our analysis, not only as markers of the extrachromosomal form of HPV, but also for the role of E2 in the viral life cycle. The E2 protein negatively regulates the expression of viral oncoproteins E6/E7, the main drivers of viral carcinogenesis, and is also important together with E1 protein for viral replication.

Methylation of CpGs in E2BSs has been shown, in vitro, to affect the binding affinity of the E2 protein, to inhibit its repressive function, and to enhance the expression of the E6 and E7 oncoproteins [29]. Higher methylation levels in E2BS3-4 have been found in CC with extrachromosomal HPV DNA [19,28]. Among the OPC samples, three groups with different methylation levels of E2BSs have been identified; complete methylation of E2BS3 and -4 have been associated with the presence of integrated HPV genomes with an intact E2 gene. In these samples, multiple viral integrated copies were present (tandem integration). An intermediate methylation level (20%–80%) was identified in samples with an extrachromosomal form of the HPV genome, and no methylation was detected in samples with the integrated HPV genome with a disrupted E2 gene [31]. Compared to previous studies, our results showed significantly higher methylation levels of the repressive E2BS2-4 in the samples with the integrated HPV genome where no E2 mRNA was detected, but the level of methylation of LCR in our study did not affect the level of expression of viral E6/E7 mRNA. A similar observation was made by Park et al. (2011) [41]. This can be explained by the results of Hatano et al. (2017), who found that the methylation of the HPV genome and human region near the integration site display the same methylation pattern [42]. So, in comparison to the extrachromosomal form of the viral genome, the methylation level of the integrated form is likely to be more influenced by the site of integration. Moreover, in the present study, the analyzed level of methylation was restricted to E2BSs, and therefore we cannot exclude the possibility that E6/7 oncogene expression is influenced by altered methylation at other HPV genome regions.

The E2 binding affinity to repression sites in LCR may be also affected by mutations. We found no mutation within E2BSs in any sample harboring either the integrated or extrachromosomal form of the HPV genome. The sequence variation in the YY1 binding site was observed in both groups tested without any difference between groups. However, sequence analysis revealed one frequent polymorphism (7518 G to A) in the binding site for the YY1 transcription factor acting as a repressor of HPV P97 transcription [43]. This polymorphism was previously found in CC samples more frequently in comparison to HPV16 isolates from healthy women [44]. We evaluated the E6*I mRNA level in tonsillar tumor samples with or without G to A transition, and detected no significantly higher transcription levels in samples with the variant type of 7518 nucleotide. Therefore, the influence of sequences variation in the YY1 sites on transcription of the viral genome needs to be further explored.

The whole E2 gene was identified in the majority of our samples. No mutation leading to protein truncation was found. Most of samples belongs to the A variant lineage and only one sample to the D variant, which was determined by comparison with the HPV16 reference sequence both for the A and D lineages. No specific variants that may represent the other mechanisms of E2 inactivation were identified in samples with the extrachromosomal form of the HPV genome. 

We observed no statistically significant difference in the viral load between the extrachromosomal/mixed and integrated genome status. The viral load was slightly, although not significantly, higher in samples with the extrachromosomal/mixed genome form. Similar data were reported by researchers examining CC [19,45,46] or HNC [16,46,47], but other studies did not find any differences in the viral loads [19,23]. When comparing the levels of expression of E6 transcripts, the E6*I transcript was more abundant than the E6 FL transcript. No increase of E6/E7 mRNA was observed in those samples with the integrated HPV genome without E2 mRNA, which is in agreement with the results of other studies on CC and HNC cancer [23,24,47]. Increasing levels of the E6/E7 transcripts were detected in premalignant cervical lesions, with the highest level being reported in invasive cancers [48], and this level seems not to be influenced by other factors, and to be sufficient for the maintenance of the malignant phenotype.

## 5. Conclusions

Taken together, our results did not confirm any of the possible speculative mechanisms of the inhibition of E2BSs in HNC with the extrachromosomal form of the virus; no mutations were detected in the E2BSs; furthermore, no difference was found in the expression of the viral oncogenes or in the viral load between samples with these two types of viral genome status. Methylation of the E2BSs was higher in tumors with the integrated form of the virus. The expression of viral oncogenes was detected in all tumors, with no preference for any of the viral oncogene transcripts tested. In line with other studies, our results show a constant expression of E6 and E7 in HNC, and the level of expression seems not to be influenced by methylation in the regulatory region or by the viral load. Therefore, we conclude that in tumors with the extrachromosomal form of HPV the expression of viral E6/E7 oncogene alone is sufficient for the induction and/or maintenance of the malignant phenotype in oropharyngeal cancer. 

## Figures and Tables

**Table 1:** HPV16 integration sites in the host genome. Identified by the APOT assay, E2 breakpoint mapping and PCR E2 in OPC samples with integrated or mixed viral genome.

		APOT Assay			E2 Breakpoint Mapping						
Sample	Status	Chromosome	Gene Code	Region in Genome	E6 mRNA	E7 mRNA	E7-E4 mRNA	E2 mRNA	E2/E5 mRNA	E5 mRNA	E2 DNA
ORL 111	Integrated	1q32	−	Intergenic area	+	+	−	−	−	−	−
ORL 125	Integrated	20p12.1	*MACROD2*	Intron	+	+	+	+	−	−	+
ORL 128	Integrated	−	−	−	+	+	−	−	−	−	+
ORL 133	Integrated	8p23.2	*CSMD1*	Intron	+	+	−	−	−	−	+
ORL 160	Integrated	17q23.1	*TUBD1*	Exon	+	+	−	−	−	−	−
ORL 126	Mixed	5q35	*CANX*	3′UTR	+	+	+	+	+	+	+
ORL 187	Mixed	13q14	*TSC22D1*	Intron	+	+	+	+	+	+	+
ORL 243	Mixed	3q28	*TP63*	Exon	+	+	+	+	+	+	+
ORL 244	Mixed	19p13.2	*CDC37*	5′UTR	+	+	+	+	+	+	+
ORL 104	Extrachromosomal	−	−	−	+	+	+	+	+	+	+
ORL 116	Extrachromosomal	−	−	−	+	+	+	+	+	+	+
ORL 137	Extrachromosomal	−	−	−	+	+	+	+	+	+	+
ORL 155	Extrachromosomal	−	−	−	+	+	+	+	+	+	+
ORL 161	Extrachromosomal	−	−	−	+	+	+	+	+	+	+
ORL 181	Extrachromosomal	−	−	−	+	+	+	+	+	+	+
ORL 257	Extrachromosomal	−	−	−	+	+	+	+	+	+	+
ORL 265	Extrachromosomal	−	−	−	+	+	+	+	+	+	+
ORL 280	Extrachromosomal	−	−	−	+	+	+	+	+	+	+

**Table 2:** Methylation analysis results. Numbers of methylated (M) and unmethylated (U) sequences for each CpG of different E2BSs. Samples are divided according to their HPV genome status into extrachromosomal, mixed and integrated. Excluded from analysis were sequences of samples ORL187 (CpG 7452 and 7460) and ORL125 (CpG 7859, 37, 43, 52 and 58) due to low number of sequences analyzed (<200 sequences).

		E2BS1				E2BS2		E2BS3				E2BS4			
HPV Genome	Position	7452		7460		7859		37		43		52		58	
	Samples	M	U	M	U	M	U	M	U	M	U	M	U	M	U
**Integrated**	ORL 111	13	2283	12	2284	1	2591	2582	17	2583	12	2586	14	2590	11
	ORL 125	17	3166	10	3173	1	97	1	100	1	100	1	100	1	100
	ORL 128	37	3081	34	3083	1	2332	5	2341	74	2272	6	2338	13	2331
	ORL 133	668	2051	642	2077	159	2499	1505	1177	1650	1032	1596	1086	1426	1256
	ORL 160	2397	3	2400	2	1	1950	1	1958	2	1957	6	1593	1	1958
**Mixed**	ORL 126	36	2624	30	2630	1	2936	33	2920	25	2926	43	2910	9	2944
	ORL 187	1	0	1	0	222	1054	1181	121	1185	117	1179	123	1185	117
	ORL 243	26	2486	21	2491	219	49	1	271	2	270	1	271	1	271
	ORL 244	94	2154	92	2157	1	2671	2246	445	2274	417	312	2378	131	2560
**Extrachromosomal**	ORL 104	N/A	N/A	N/A	N/A	N/A	N/A	N/A	N/A	N/A	N/A	N/A	N/A	N/A	N/A
	ORL 116	123	1106	102	1127	22	1241	791	484	805	469	797	477	850	425
	ORL 137	150	3120	56	3214	1	1403	14	2402	16	2399	14	2402	1	2415
	ORL 155	10	2495	9	2496	1	2399	3	2413	6	2409	1	2415	2	2414
	ORL 161	15	2530	11	2534	1	2281	9	2286	6	2289	10	2285	4	2291
	ORL 181	52	1284	32	1305	7	1588	14	1588	1	1602	34	1569	27	1576
	ORL 257	14	2035	13	2036	1	1933	2	1945	2	1945	4	1943	5	1942
	ORL 265	25	2217	7	2235	2	1773	1	1788	4	1785	17	1772	1	1788
	ORL 280	28	2462	22	2468	2	3247	20	3234	23	3230	22	3232	106	3148

**Table 3:** Detection of nucleotide variation in the LCR region. Nucleotide positions of variations in samples as compared to HPV16 reference sequence (GenBank: K02718.1). Sample ORL 104 was matched to the HPV16 D3 lineage sequence (GenBank: AF402678). Affected transcription factor (TF) binding sites are also marked, i.e., YY1, Tef1 and NF1.

	Nucleotide Position	7482	7486	7518	7629	7666	7686	7712	7726	7740	7761	7783	7853	7883	7888	12	13	24	28	109	131	145	188
**Integrated**	ORL 111																						
	ORL 125			A												C			A		G		
	ORL 128			A				G															
	ORL 133			A																			
	ORL 160			A																			
**Mixed**	ORL 126			A																			
	ORL 187			A																			
	ORL 243																T						C
	ORL 244			A																			
**Extrachromosomal**	ORL 116																						
	ORL 137			A																			
	ORL 155			A																			
	ORL 161			A																			
	ORL 181			A	A																		
	ORL 257			A																			
	ORL 265			A														G		C			
	ORL 280												A										
	ORL 104	C	A	A		T	A		C	G	T	T		T	T							T	
**AF402678**		C	A	A		T	A		C	G	T	T		T	A							T	
**K02718.1**		A	G	G	C	C	C	T	A	T	C	C	G	C		T	C	C	G	T	A	G	G
**TF**				YY1			Tef1	NF1				YY1											

## Supplementary Materials

The supplementary materials are available online at https://www.mdpi.com/1999-4915/12/1/42/s1.
