# Supplementary material for: Detailed Characteristics of Tonsillar Tumors with Extrachromosomal or Integrated Form of Human Papillomavirus
Source: Viruses. 2019 Dec 30;12(1):42. doi: 10.3390/v12010042 (PMC7019694; doi:10.3390/v12010042)
Supplement: Supplementary file 1 [file viruses-12-00042-s001.pdf]

Supporting information

Table S1- List of primers used for mapping of E2 integration breakpoint.

Amplicon	Primer direction	Sequence (5'-3')	Position
**E7**	forward	AGCACACACGTAGACATTCGTA	772-793
	reverse	AGATGGGGCACACAATTCCT	841-822
**E7-E4**	forward	AGCACACACGTAGACATTCGTA	772-793
	reverse	GGTGTCTGGCTCTGATCTTG	3496-3477
**E2B**	forward	ATGCATTATACAAACTGGA	3140-3158
	reverse	TGCACAAAATATGTTCGTATTCC	3264-3242
**E2**	forward	ATTATTAGGCAGCACTTG	3383-3400
	reverse	GGTGTCTGGCTCTGATCTTG	3496-3477
**E2/E5**	forward	GATAGTGAATGGCAACGTGAC	3767-3787
	reverse	GATGCAGTATCAAGATTTG	3872-3854

E2 protein	Transactivation/repression domain								Hinge region										DNA-binding /dimerization domain		
	35	64	126	135	142	143	157	165	203	206	208	210	211	219	232	254	270	271	310	341	344
ORL 104	Q			K	D	T	I	Q	D		A		T	S	K	N		V	K	C	E
**AF402678**	Q			K	D	T	I	Q	D		A		T	S	K	N		V	K	C	E
ORL 116										F^{NP}											
ORL 125												T*		S*					K*		
ORL 126			Y^{P}									T*		S*					K*		
ORL 128														S*							
ORL 133												T*		S*					K*		
ORL 137														S*							
ORL 155														S*							
ORL 161											S^{P}			S*							
ORL 181														S*							
ORL 187														S*							
ORL 243		I^{NP}																			
ORL 244 - h														S*							
ORL 244 - l														S*			G^{NP}	L^{NP}			
ORL 257														S*							
ORL 265														S*							
ORL 280																					
**K02718.1**	**H**	**V^{NP}**	**C^{NP}**	**T**	**E**	**A**	**L**	**R**	**N**	**S^{P}**	**P^{NP}**	**I***	**I**	**P***			**A^{NP}**	**F^{NP}**	**T***		

Table S2 Detection of protein variation in E2. Protein polymorphism in samples as compared to HPV 16 reference sequence (GenBank: K02718.1). Sample ORL 104 was matched to HPV 16 D3 lineage sequence (GenBank: AF402678). NP: non polar, P: polar amino acid, *polymorphism in position 210, 219 and 310 were also describe in https://www.uniprot.org/uniprot/P03120/protvista
